# Gene model for the ortholog of *gfzf* in *Drosophila cardini*

**DOI:** 10.17912/micropub.biology.002296

**Published:** 2026-08-10

**Authors:** Brianna Lowry, Pablo Chialvo, Clare Scott Chialvo

**Affiliations:** 1 Biology, Appalachian State University, Boone, North Carolina USA

## Abstract

We developed a gene model for the *GST-containing FLYWCH zinc-finger protein* ortholog ( *gfzf* ) in the ASM1890373v1 Genome Assembly (GenBank Accession: GCA_018903735.1 ) of *Drosophila cardini* . This ortholog was characterized as part of a developing dataset for a comparative study of detoxification gene family evolution in the *immigrans* - *tripunctata* radiation of the genus *Drosophila* using an adapted Genomics Education Partnership gene annotation protocol for Course-based Undergraduate Research Experiences.

**Figure 1. Genomic neighborhood and gene model for gfzf ortholog in D. cardini :** (A) **Synteny comparison of the genomic neighborhoods for *gfzf* in *Drosophila melanogaster* and *Drosophila cardini* .** Thin underlying arrows indicate which DNA strand the target gene, *gfzf* , is located on in *D. melanogaster* (top) and *D. cardini* (bottom). The thin arrows pointing to the left indicate that *gfzf* is on the negative strand in both *D. melanogaster* and *D. cardini* . The wide gene arrows pointing in the same direction as *gfzf* are on the same strand relative to the thin underlying arrows, while wide gene arrows pointing in the opposite direction of *gfzf* are on the opposite strand relative to the thin underlying arrows. White gene arrows in *D. cardini* indicate orthology to the corresponding gene in *D. melanogaster* . Other colors of arrows indicate: black = non-orthology and grey = present in both neighborhoods but not syntenic. Gene symbols given in the *D. cardini* gene arrows indicate the orthologous gene in *D. melanogaster* , while the locus identifiers are specific to *D. cardini* . (B) **Gene Model in GEP UCSC Track Data Hub** (Raney et al., 2014). The coding-regions of *gfzf* in *D. cardini* are displayed in the User Supplied Track (red); coding sequences (CDS) are depicted by thick rectangles and introns by thin lines with arrows indicating the direction of transcription. Subsequent evidence tracks include Spaln of *D. melanogaster* Proteins (purple, alignment of Ref-Seq proteins from *D. melanogaster* ), Coding Regions Predicted by Augustus (dark blue), GeMoMa (teal), and NSCAN PASA-EST (dark green), and RNA-Seq from mixed sex adult flies (brown; alignment of Illumina RNA-Seq reads from *D. cardini* – Erlenbach et al., 2023). (C) **Dot Plots of gfzf-PB and gfzf-PD in *D. melanogaster* ( *x* -axis) vs. the orthologous peptides in *D. cardini* ( *y* -axis).** Amino acid number is indicated along the left and bottom; CDS number is indicated along the top and right, and CDSs are also highlighted with alternating colors. Line breaks in the dot plot indicate areas of with low sequence similarity between species. In the gfzf-PB dot plot, there is a series of three small breaks in the dot plot towards the beginning of CDS 2 (purple box – a). There is also a small break at the end of CDS 2 (light blue box – b). The gfzf-PD dot plot contains two small breaks in CDS 2 (green box – c and yellow box – d) (D) **Idiosyncrasies in protein alignment.** In gfzf-PB, CDS 2 contains four small breaks. The first set of three consecutive short breaks in CDS 2 (purple box - a) extends from amino acid 274 – 579 approximately. This span contains 3 areas of short insertions into the *D. cardini* gene model or deletions from the ortholog in *D. melanogaster* . Beyond these changes, most amino acids in these regions are similar. The fourth break in the gfzf-PB model occurs at the end of CDS 2 (light blue box - b) from amino acid 720 – 795 approximately. This gap is due to three areas of insertion in the ortholog in *D. cardini* . The gfzf-PD model includes two small breaks in CDS 2. The first break spans a region of 17 amino acids (77-94), which are all similar except for two amino acids (green box - c). The second break covers nine amino acids (82-90) and is also due to two dissimilar amino acids (yellow box - d).

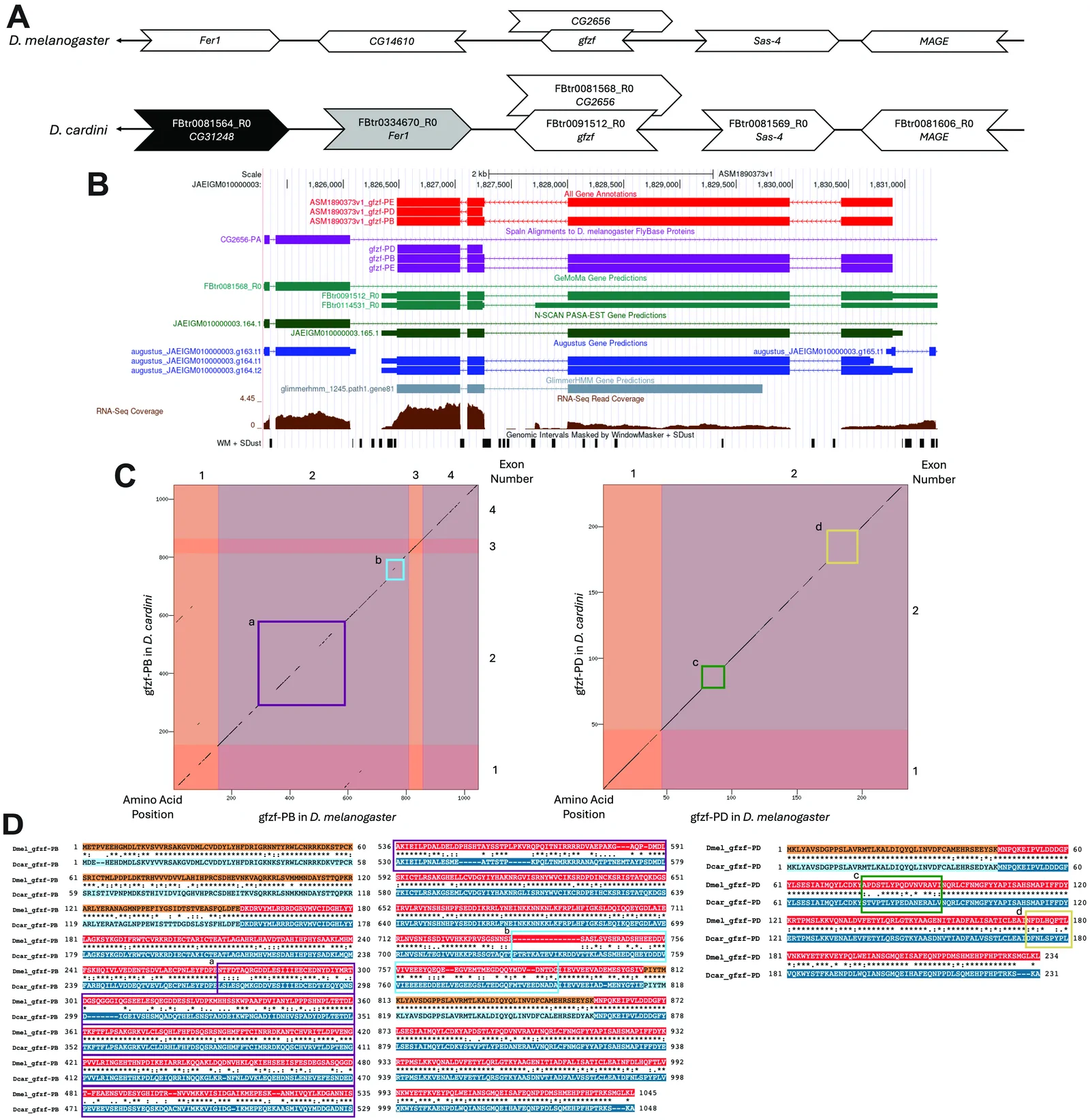



## Description

**Table:** 

*This article reports a predicted gene model generated by undergraduate work using a structured gene model annotation protocol defined by the Genomics Education Partnership (GEP; thegep.org) for Course-based Undergraduate Research Experience (CURE). The following information in quotes may be repeated in other articles submitted by participants using the same GEP CURE protocol for annotating Drosophila species orthologs of Drosophila melanogaster detoxification genes.* “Within insects, the process of detoxifying xenobiotics and host secondary metabolites is a three-phase process that involves functionalization, conjugation, and excretion of these compounds. Expansions of known detoxification gene families ( *e.g.* , cytochrome P450s) are associated with diet breadth and insecticide resistance (Ranson et al., 2002; Després et al., 2007; Rane et al., 2016). With the increasing availability of high-quality genomes for non-model organisms, including *Drosophila* species beyond *D. melanogaster* , it is now possible to perform large scale comparative studies (Robinson et al., 2011; Kim et al., 2021; Threfall and Baxter, 2021). Careful manual annotation and curation of gene models can improve upon computational gene predictions in non-model species, which aids the accuracy of studies on gene and genome evolution (Mudge and Harrow, 2016; Tello-Ruiz et al., 2019). To aid in these annotations, the Genomics Education Partnership (thegep.org) developed a curriculum involving web-based tools that allow undergraduates to engage in authentic course-based research focused on manually annotating genes in non-model species (Rele et al., 2023). The orthologous gene models, including the one presented here, then provide a reliable basis for further evolutionary genomic analyses when made available to the scientific community. The gene ortholog described here in *Drosophila cardini* for *GST-containing FLYWCH zinc-finger protein* ( *gfzf* ), a member of the glutathione S-transferase (GST) was characterized as part of a developing dataset for a comparative study of detoxification gene families in the *immigrans* - *tripunctata* radiation of the genus *Drosophila* .” (Williams et al., 2026) “In the subgenus *Drosophila* , *D. cardini* Sturtevant 1916 is a member of the *cardini* subgroup in the *cardini* species group of the *immigrans-tripunctata* radiation (Heed and Krishnamurthy, 1959; Bächli, 2005). Species in the *cardini* subgroup are found in the mainland Neotropics, and the range of *D. cardini* extends from Florida to Brazil (Heed, 1962). Members of the *cardini* group primarily feed and develop on fruit and flowers (Markow and O'Grady, 2008). However, *D. cardini* is also reported to feed on mushrooms and can tolerate the cyclopeptide toxin α-amanitin (Stump et al., 2011).” (Patel et al., 2026) “One class of phase II detoxification enzymes are the glutathione S-transferases (GSTs), which act by conjugating xenobiotics or products of phase I detoxification with a glutathione to make them more hydrophilic prior to excretion (Enayati et al., 2005). GSTs are classified into two families based on their cellular location (microsomal and cytosolic). Within insects, cytosolic or canonical GSTs are divided into six classes (delta, sigma, epsilon, zeta, theta, and omega; Enayati et al., 2005; Ketterman et al., 2011).” (Williams et al., 2026) *GST-containing FLYWCH zinc-finger protein* ( *gfzf* ) is classified as a biochemically active class I GST like gene (Ranson et al., 2001; Dai et al., 2004). It is unique in that it contains both a functional GST and zinc finger protein domains, which allow it to act as both a transcriptional coactivator and GST (Dai et al., 2004.; Baumann et al., 2018). Expression of this gene is suppressed by the accumulation and deposition of β-amyloid (Li et al., 2023). Beyond the fact that *gfzf* will bind to glutathione very little is known regarding its function as a GST (Dai et al., 2004; Baumann et al., 2018).

We propose a gene model for the *D. cardini* ortholog of the *D. melanogaster* *GST-containing FLYWCH zinc-finger protein* ( *gfzf* ) gene. The genomic region of the ortholog corresponds to the GeMoMA prediction FBtr0091512_R0 in the ASM1890373v1 Genome Assembly of *D. cardini* ( GCA_018903735.1 – Kim et al., 2021). This model is based on mixed sex, adult RNA-Seq data from *D. cardini* (Erlenbach et al., 2023; https://doi.org/10.5061/dryad.hdr7sqvq2) and *gfzf* in *D. melanogaster* using FlyBase release FB2024_02 ( GCA_000001215.4 ; Gramates et al., 2022; Jenkins et al., 2022; Larkin et al., 2021).

***Synteny***

The reference gene, *gfzf ,* occurs on chromosome 3R in *D. melanogaster* . It is nested in *CG2656* , flanked upstream by *Spindle assembly abnormal 4* ( *Sas-4* ) and *MAGE* , and flanked downstream by *CG14610* and *48 related 1* ( *Fer1* ). The *tblastn* search of *D. melanogaster* gfzf-PB (query) against the *D. cardini* (GenBank Accession: GCA_018903735.1 Genome Assembly (ASM1890373v1)) placed the putative ortholog of *gfzf* within contig_2098 (JAEIGM010000003.1) which corresponds to the GeMoMa prediction FBtr0091512_R0 (E-value: 0.0; percent identity: 68.74% as determined by *blastp* ). The putative ortholog is nested in GeMoMa gene prediction FBtr0081568_R0, which corresponds to *CG2656* in *D. melanogaster* (E-value: 0.0; identity: 87.99%). It is flanked upstream by GeMoMa gene predictions FBtr0081569_R0 and FBtr0081606_R0, which correspond to *Sas-4* and *MAGE* in *D. melanogaster* (E-value: 0.0 and 2e-85; identity: 66.38% and 56.83%, respectively, as determined by *blastp* ; Figure 1A ; Altschul et al., 1990). The putative ortholog of *gfzf* is flanked downstream by GeMoMa predictions FBtr0334670_R0 and FBtr0081564_R0, which correspond to *Fer1* and *CG31248* in *D. melanogaster* (E-value: 4e-147 and 4e-123; identity: 79.06% and 62.95%, respectively, as determined by *blastp* ). The putative ortholog assignment for *gfzf* in *D. cardini* is supported by the following evidence: The *tblastn* results are of good quality, and all coding sequences (CDS) and isoforms found in *D. melanogaster* also appear to be present in *D. cardini* . The local synteny for *gfzf* ortholog is mostly conserved. The gene predictions in *D. cardini* for the target gene, the gene it is nested within ( *CG2656* ), and the two upstream neighboring genes ( *Sas-4* and *MAGE* ) are orthologous with the neighborhood in *D. melanogaster* . The two downstream gene predictions in *D. cardini* correspond to *Fer1* and *CG31248* , which are the second and fourth downstream genes in *D. melanogaster* . Given the quality of the blastp results and the fact that all predictions are either syntenic or correspond to genes that are in close proximity to *gfzf* in *D. melanogaster* , we conclude that the GeMoMa gene prediction FBtr0091512_R0 is an ortholog of *gfzf* in *D. cardini* ( Figure 1A ).

***Protein Model***

The *gfzf* ortholog in *D. cardini* has four CDSs within the genome sequence, and it encodes two unique protein sequences. The first unique protein sequence is translated from two messenger RNA (mRNA) isoforms consisting of four CDSs that differ in their untranslated regions (gfzf-RB and gfzf-RE; Figure 1B ). The second unique protein sequence is translated from a single mRNA composed of two CDSs (gfzf-RD; Figure 1B ). Relative to the ortholog in *D. melanogaster* , the CDS number and protein isoform count are conserved *.* The protein sequence of gfzf-PB in *D. cardini* has 68.3% identity (80% similarity) with the protein-coding isoform gfzf-PB in *D. melanogaster* , as determined by *blastp* ( Figure 1C ). A comparison of the protein sequence of gfzf-PD in *D. cardini* to the isoform in *D. melanogaster* finds 82.5% identity and 91.0% similarity. These levels of divergence are not surprising given that *D. melanogaster* and *D. melanogaster* belong to two separate subgenera ( *Drosophila* and *Sophophora* respectively) that diverged approximately 45-60 MYA (Russo et al., 1995; Tamura et al., 2004; Obbard et al., 2012). Coordinates of this curated gene model are archived in the CaltechDATA repository (see “Extended Data” section below).

## Methods

The annotation methods used in this project are adapted from those described in Rele et al. (2023), which includes algorithms, database versions, and citations for the complete annotation process developed for the Pathways Project. The methods for the current project are detailed in brief below with notes on significant differences between this protocol and the one described in Rele et al. (2023). The students use the GEP instance of the UCSC Genome Browser v.435 (https://gander.wustl.edu ; Kent et al., 2002; Raney et al., 2024) to examine the genomic neighborhood of their reference detoxification gene in the *D. melanogaster* genome assembly (Aug. 2014; BDGP Release 6 + ISO1 MT/dm6). Students obtain the protein sequence for the *D. melanogaster* target gene for a given isoform and use a *tblastn* search of the sequence against their target *Drosophila* species genome assembly ( *D. cardini* ( GCA_018903735.1 – Kim et al., 2021)) on the NCBI BLAST server (https://blast.ncbi.nlm.nih.gov/Blast.cgi, Altschul et al., 1990) to identify the putative ortholog location. Students compare the genomic neighborhood of the putative ortholog to that of the reference gene in *D. melanogaster* . This local synteny analysis includes a minimum of two upstream and downstream genes relative to the potential ortholog. As no RefSeq protein data is available for these species, comparisons are based on gene predictions that correlate with gene expression data in the putative ortholog neighborhood. Using the multiple alignment tracks feature in the Genome Browser, students examine other sets of genomic evidence, including Spaln alignment of *D. melanogaster* proteins, multiple gene prediction tracks (e.g., GeMoMa, Augustus, NSCAN PASA-EST), and mixed sex RNA-Seq adult expression data from the target species generated by Erlenbach et al. (2023; https://doi.org/10.5061/dryad.hdr7sqvq2). Information on the genomic structure information (e.g., CDSs, intron-exon number, number of isoforms) for the reference gene in *D. melanogaster* is retrieved using Gene Record Finder (https://gander.wustl.edu/~wilson/dmelgenerecord/index.html; Rele et al *.,* 2023). To determine approximate splice sites within the target gene, a *tblastn* search using the CDSs from the *D. melanogaste* r reference gene against the putative ortholog location (10kb up- and downstream of the target gene prediction). Coordinates of the CDS(s) are refined by examining aligned RNA-Seq data, identifying canonical splice site sequences, and ensuring the maintenance of an open reading frame. Students confirm the biological validity of their target gene model using the FlySeq Gene Model Checker (https://gander2.wustl.edu/~wilson/genechecker-flyseq/), which compares the hypothesized target gene model's structure and translated sequence against the *D. melanogaster* reference gene. At least two independent models for this gene are generated. These models are reconciled by a third independent researcher to produce the final model presented here. Note: comparison of 5' and 3' UTR sequence information is not included in this GEP CURE protocol.

## Data Availability

Description: Zipped archive containing FASTA, PEP, and GFF of gfzf model. Resource Type: Dataset. DOI: https://doi.org/10.22002/c79h5-cfe79
